# A Network Centrality Method for the Rating Problem

**DOI:** 10.1371/journal.pone.0120247

**Published:** 2015-04-01

**Authors:** Yongli Li, Paolo Pin, Chong Wu

**Affiliations:** 1 School of Business Administration, Northeastern University, Shenyang 110819, P.R.China; 2 Dipartimento di Economia Politica e Statistica, Università degli Studi di Siena, 53100 Siena, Italy; 3 School of Management, Harbin Institute of Technology, Harbin 150001, P.R.China; Centre de Physique Théorique, FRANCE

## Abstract

We propose a new method for aggregating the information of multiple users rating multiple items. Our approach is based on the network relations induced between items by the rating activity of the users. Our method correlates better than the simple average with respect to the original rankings of the users, and besides, it is computationally more efficient than other methods proposed in the literature. Moreover, our method is able to discount the information that would be obtained adding to the system additional users with a systematically biased rating activity.

## 1 Introduction

When many reviewers rate goods or projects, the exercise of aggregating all this information is a useful one, especially for big online sellers, when reporting huge feedback data from consumers. Being able to offer a reliable aggregate ranking benefits the consumers to recognize favorite goods, and it is a service provided e.g. by Amazon (www.amazon.com), Ebay (www.ebay.com) and Taobao (www.taobao.com). Moreover, it is actually the core of the service of other online sites, such as Tripadvisor (www.tripadvisor.com). Many works in the recent past have demonstrated the significance of scores and remarks given by the online shoppers: some of them are [1, 2, 3, 4].

Given a set of items, a set of users and the rating scores of each user–item pair, the general *rating problem* is to rate each item with a single comprehensive score. In this context, this paper aims to present a reasonable *rating method*, implementable in efficient polynomial time by an algorithm, and whose results can be in accordance with most of customers’ rankings. A strictly related problem is the one of *collaborative filtering* in *recommender systems*, where an algorithm tries to extrapolate missing information about the items from the rating activity of the users, in order to provide a specific ad–hoc ranking for each users also on the items that she has not rated (on this see [5, 6, 7, 8] discuss how to aggregate the information from multilayer networks, while [9] show the importance of centrality measures for this problem). We discuss in the conclusion how our method could be adapted to this setting.

The commonly adopted rating method in those real world applications is the averaging, which is implementable in linear time, but has been found defective. In particular, the result of the averaging is likely to violate most customers’ preferences, not only because of the *Arrow impossibility theorem* [10], but also when a coherent ranking is actually available (as in Example 2.6 of this paper—see [11] for a recent analysis of this theoretical problem). On the other hand, more complicated methods must take into account other constraints. One is that in many cases most of the users rate only a small fraction of all the available items, and it becomes useful to consider also the weight of all this missing information. Another one is that the computational complexity of every aggregate rating must be taken seriously into account, because the numbers of items and users can be hundreds of thousands. So, it is important to find a good trade off that is at the same time efficient in approaching a good correlation with most of cutomers’ preferences, but is also actually implementable by the online sites.

Another important issue to be considered in the online rating applications is the problem of fake data. In some cases, true or fake users could be maliciously biased in favor of some specific items. This can happen because single fake accounts, known as *Sybils* (see [12, 13]), are created; or many of them are systematically included in the system to induce an intentionally biased evaluation (a phenomenon called *Crowdturfing*, see [14]). In such cases the simple rating method of averaging is considered as a much better solution than the methods that preserve ordering, as averaging can be thought as an unbiased estimator of the *true value* of the items when evaluations are affected by some *white noise* (on this, see [15]). However, if the additional fake users are all coordinated in favor of or against to some items, then this added noise will clearly not have an average null effect on the final average, and a ranking method should be able to discount the information that comes from systematically biased users.

We propose a new rating method, which we call the *network centrality* method. In a setting where the set of available scores is finite, we consider a network where each node is a score for one item, i.e. a couple score–item, and there is a link between two nodes if there is at least one user that ranked the two items with exactly those two scores. Looking at the centrality of the nodes in this network, we find what are the score–item couples that are more *coherent* with rankings of the users. We do so borrowing from the literature on complex networks that have analyzed the importance of the *centrality* of a node. We adopt the Katz–Bonacich spectral measure of centrality (see [16] for a fairly recent exposition) to define a *network centrality* method of aggregate ranking (see Bramoullé et al. (2014) for a recent discussion of the applications of the Katz–Bonacich centrality to economic environments with peer–effects). The intuition behind the effectiveness of our method is that a couple score–item gets recursively more weight the more weight have other score–item couples linked to it. Thus, since the link between those couples are created by some users, those users become more authoritative sources on those items. In this way, our method approaches a *good* correlation with most of costumers rating, in a recursive way that is typical to all spectral measures of centrality: score–item couples are more central when more users link them to other central score–item couples.

Our network centrality method results to have some desirable features. First, it is computationally efficient, if compared to other measures in the literature. Second, it performs very well in maintaining most of the original rankings of the reviewers, both on randomly generated data, and on real data from an online rating platform. Third, it is robust to the artificial insertion of users systematically providing fake data.

The rest of the paper is organized as follows. In Section 2 we provide a motivating intuition for our method, and describe it formally. In Section 3 we report results of an extensive numerical analysis that compares our method with others from the existing literature. In Section 4 we apply our method to a real online dataset where people rate movies. Section 5 concludes.

## 2 Intuition and theory for our approach

Consider a finite set of items and a finite set of users. In a setting where each user evaluates an independent subset of the items, the *rating problem* consists of aggregating all this information in a single vector by assigning a score to each item. A *rating method* is an algorithm that provides a solution. We consider different *measures* to evaluate rating methods and discuss their computational efficiency.

### 2.1 Intuition

Let us start with an example that provides the intuition for our method. In a rating environment where users assign marks (from 1 to 5) to items, consider the situation illustrated in Table 1.

**Table 1:** A simple example.

	*Users*			
*Items*	1	2	3	4
*a*	2	–	4	–
*b*	1	1	5	5
*c*	–	2	–	4

**Fig 1:**
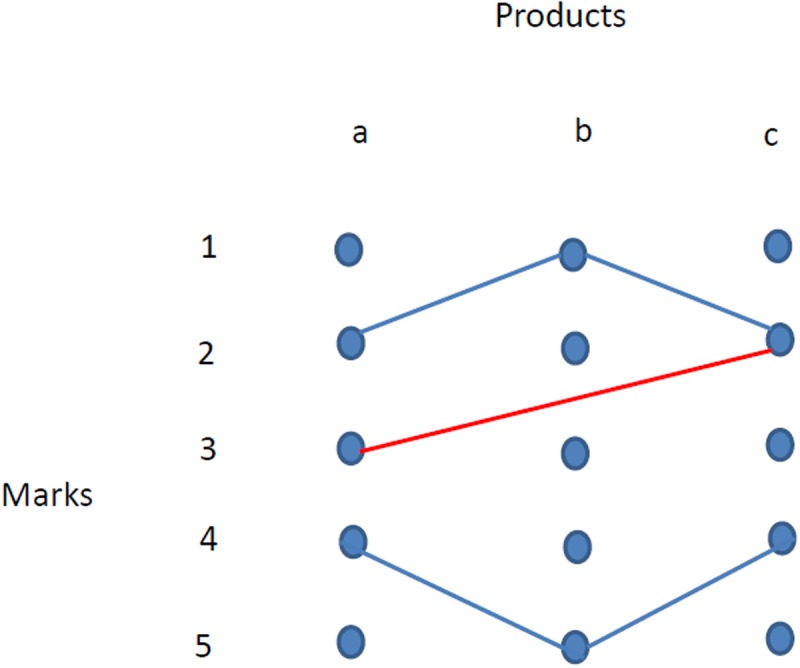
The network representation of Table 1 (the blue lines) and Table 2 (including the red line).

What is the score that we should attribute to product *b* from this table? The situation here is fully symmetric between two users attributing a mark of 1, and the other two attributing a mark of 5. This symmetry is not only in the grades they attribute to good *b*, but also in the overall markings through all products, as depicted by the blue lines in Fig. 1: this is a network where nodes are all possible marks for all goods, and there is a link between two nodes if at least one user gave those specific two marks to those two goods.

Now suppose that a new user 5 enters and assigns marks only to items *a* and *c*, as depicted in Table 2, where a new column has been added. Apparently this adds no information on the value of item *b*. However, if we look at Fig. 1 this breaks symmetry: now this new user (the red link) agrees on item *c* with an agent that gave a low mark to item *b*. If we want to assign some value to this new piece of information, we will give more weight to mark 1 for item *b*, or equivalently a higher weight to node *b*1 in the network of Fig. 1.

**Table 2:** One more column for Table 1.

	*Users*				
*Items*	1	2	3	4	5
*a*	2	–	4	–	3
*b*	1	1	5	5	–
*c*	–	2	–	4	2

### 2.2 Formal Model

In general, a rating problem can be formalized and solved in the following way. Consider *M* items that receive marks from 1 to *a* ∈ ℕ, from *N* users. A user *n* will typically assign marks only to a subset of *k*_*n*_ out of the *M* items, and consequently assign no mark (the − symbol) to all the other *M* − *k*_*n*_ items. We say that *xi* ∈ *S*_*n*_ if user *n* assigns mark *i* to item *x*. This environment can be represented by an *N* × *M* matrix with elements from {1, …, *a*}∪{−}, which will typically be sparse (i.e. with many ‘−’ elements). We call **r** this matrix, so that *r*_*i*, *m*_ is the mark (or the − symbol) that user *i* assigns to items *m* (we will come back to this representation when analyzing other ranking measures in the literature, in Section 2.4). Another way to represent this environment is with an undirected weighted network with *a* ⋅ *M* nodes (one for every possible non–blank mark for each product) and where links are weighted in the following way. The weight of a link between the node *xi* and a different node *yj* is given by the formula

$$\begin{array}{c} \ell_{xi,yj}\equiv \sum_{n\in N}\frac{1}{k_{n}-1}\left({𝕀}_{xi\in S_{n}}\cdot {𝕀}_{yj\in S_{n}}\right)\;\;, \end{array}$$ (1)

where 𝕀_*x* ∈ *X*_ is the indicator function that has value 1 if *x* is an element of *X*, and has value 0 otherwise. We set by definition ℓ_*xi*, *xi*_ = 0, so that there are no links from any node to itself. What formula (1) says is that if a user *n*, who already marked *k*_*n*_ items, assigns mark *i* to item *x*, then this will add a value of $\frac{1}{k_{n}}$ to each link between node *xi* and all those nodes *yj* already assigned by user *n* and present in the set *S*_*n*_. Algebraically this just adds an aggregate value of 1 to all the links of node *xi*. We call *L* the symmetric *aM* × *aM* *adjacency matrix* obtained from (1).

If we sum any row or column of this matrix, say the one labelled *xi*, the result is

$$\begin{array}{c} \sum_{yj\neq xi}\sum_{n\in N}\frac{1}{k_{n}-1}\left({𝕀}_{xi\in S_{n}}\cdot {𝕀}_{yj\in S_{n}}\right)=\sum_{n:\;xi\in S_{n}}\frac{k_{n}-1}{k_{n}-1}=\left|\left\{n:\;xi\in S_{n}\right\}\right|\;\;, \end{array}$$

where the first passage is due to the fact that each user rating *xi* puts node *xi* in relation with other *k*_*n*_ − 1 nodes. So, the sum on each row or column of matrix *L* is just the number of users that actually rated good *x* with mark *i*. In other words, in this network the *degree* of a node is just the amount of *i* marks provided to *x* by the *N* users. However, as is well known from network theory (see e.g. [17, 18, 19]), the degree of a node is only one piece of information about its role in the network structure. Going back to the example in Fig. 1, even if nodes *b*1 and *b*5 have the same degree, node *b*1 is more *central* in the network, because there are more other nodes *connected* to it.

Another way to measure centrality is to consider *network paths*. A network path is a set of links that connects indirectly two nodes. In the network representation of our rating environment, a path of length *d* is given by *d* ordered users who, pairwise, agreed on the same mark to assign to the same item. In Fig. 1, considering all links, there is a path of length 3 between *a*2 and *a*3 because user 1 picked *a*2 and agreed with user 2 on *b*1, then user 2 agreed with user 5 on *c*2, and finally user 5 picked *a*3. The fact that this path passes through *b*1 and *c*2 assigns some structural *centrality* to these two nodes. Let us be more formal, the weight of the paths of length 1 between any two nodes are represented by matrix *L* itself, those of length 2 are simply described by its square *L* ^2^, and so on, with longer paths that are exactly represented by higher powers of matrix *L*. If we call *I* the *aM* × *aM* identity matrix, and $\vec{1}$ the column vector made of *aM* ones, the Katz–Bonacich centrality $\vec{c}$ of a node is given by the implicit formula (see also [20, 21] and note [22])

$$\begin{array}{ccc} \vec{c} & = & \beta L\vec{c}\;\;. \end{array}$$

As long as *β* is not larger than the inverse of the maximum eigenvalue of *A*, this can be made explicit by its unique solution

$$\begin{array}{ccc} \vec{c} & = & \beta L\vec{1}+\beta^{2}L^{2}\vec{1}+\beta^{3}L^{3}\vec{1}+\cdots \\ & = & {\left(I-\beta L\right)}^{-1}\vec{1}-\vec{1}\;\;. \end{array}$$ (2)

Parameter *β* > 0 plays the classical role of a multiplicator factor, and tells us how much we want to decrease the weight of longer paths. Element $L\vec{1}$ in the second line of Equation (2) is exactly the vector that counts the degree of each node, while the following elements consider larger paths. As for any other spectral measure of centrality (see [19]), a node gets recursively more weigth the more weight its neighbors have, and this makes spectral centrality measures the natural candidates when we want to find an endogenous weight of importance for the nodes of a network (see also [23]). In our context, this simply tells that a specific grade for one item (i.e. a score–item node of the network) has higher weight the more users, among those that gave that score to that item, also agreed on highly weighted scores for other items.

Then, to attribute a score to product *x* one can make an average of all the possible marks for this product (i.e. *x*1, *x*2, …, *xa*), weighted by their centrality:

$$\begin{array}{c} s_{x}=\frac{\sum_{i=1}^{a}c_{xi}\cdot i}{\sum_{i=1}^{a}c_{xi}}\;\;. \end{array}$$ (3)

This score takes into account the aggregate information of the whole network and the correlations between the opinions in the overall poll of users. In this way we obtain a vector $\vec{s}$ that is our solution to the rating problem, and we call it the *network centrality* (NC) method. An important property of Equation (3) is that at the limit of *β* → 0 it coincides with the simple average, which is what would be obtained truncating the second line of Equation (2) after the first element $\beta L\vec{1}$.

### 2.3 What is the best value for *β*?

The Katz–Bonacich centrality has the additional advantage of being *tunable* with a single parameter *β*, which accounts at one extremum for the simple degree–centrality measure. Variable *β* represents the *peer effect* between neighboring judgments in the adjacency matrix *L* filled by all possible ranks for each item. The best value for *β* may clearly depend on the other variables of the problem, and in particular on the adjacency matrix. From the way it is built (Equation (1)), matrix *L* is an *aM* × *aM* symmetric matrix with all non–negative entries.

It is well known (see e.g. [24]) that the strength of the peer effect depends on the largest eigenvalue of the adjacency matrix. From the Perron-Frobenius theorem, the largest eigenvalue of *L* is its unique positive eigenvalue *λ* ^+^, that lies in the interval (0, *N*) (see note [25]).

It is possible to balance the peer effect of the network structure with some *β* that is inversely proportional to *N*, or to do it with a *β* that is inversely proportional to the actual *λ* ^+^ of matrix *L*. In the simulations of next section we try the following 6 values for *β* (in decreasing order): 1/*λ* ^+^, 1/*N*, 1/5*N*, 1/10*N*, 1/25*N* and 1/50*N*. Actually, when *β* = 1/*λ* ^+^, Equation (2) is not defined; however, at this limit $\vec{c}$ approximates the eigenvector of *L* corresponding to *λ* ^+^, and this is what we compute in this case (it is also well known, as discussed in [16], that this limiting result converges on a path that is extremely volatile to tiny fluctuations, simply because Equation (2) approximates the inversion of a singular matrix).

At the other side of the interval, namely at the limit *β* → 0 the NC method will coincide with the average. Then, we conjecture that a larger *β* would lead to a better result, but there is a trade off at 1/*λ* ^+^, which is the actual limit to stability of the infinite series in Equation (2). Because of this, we try also an intermediate value between the first two, which is 2/(*λ* ^+^ + *N*).

### 2.4 Computational efficiency

The infinite series of Equation (2) is perfectly computed in the third line, and matrix inversion is a very well studied problem. Actually, [26] has recently proposed an algorithm that computes the inverse of a general *n* × *n* matrix in *O*(*n* ^2.373^) computational time. Moreover, faster algorithms provide *good enough* approximate solutions when the original matrix is *sufficiently* sparse. On this see e.g. [27]. Note also that the infinite sum in the second line of Equation (2) can be truncated at the *i* ^*th*^ step, and so its computational cost can be arbitrarily reduced at the expenses of accuracy. In fact, at the limit *β* → 0 we have the truncation *i* = 1 and the NC method coincides with the average, whose computational cost is linear. So, our method needs to invert an *aM* × *aM* matrix, where *a* is a constant, and the matrix inversion can be solved at worst in *O*(*M* ^2.373^) computational time.

Our ranking method is not the first one that applies spectral analysis. The first method based on spectral analysis is the Analytical Hierarchy Process proposed by [28], which requires that all the users rate all the items. More in general, methods based on eigenvector centrality (as the one used by [29]), are unstable, as has been shown in the literature on peer effects in social networks above).

With a different approach, [30] proposed an algorithm to minimize the aggregate discrepancy of an overall ranking with respect to each individual’s ranking, but their algorithm is NP-hard, which makes it unfeasible for instances with many items and users. [31] propose an approximation algorithm that works in polynomial time and approximates the one of [30]. Building on that, another ranking method that has recently been proposed in the literature is the Separation–Deviation (SD) method provided in equation (8a) of [32]. In this paper we actually adopt the SD method as a comparison with respect to our method, and the objective measure they minimize as one of the benchmarks of evaluation. Here, we adapt it to our notation. They aim to find the vector $\vec{s}$ that solves the following minimization problem

$$\begin{array}{ccc} \min_{\vec{s}} & & \alpha \;\left(\sum_{m=1}^{M}\sum_{i=1}^{N-1}\sum_{j=i+1}^{N}w_{ij}^{k}{\left(s_{i}-s_{j}-r_{i,m}+r_{j,m}\right)}^{2}\right)+\left(\sum_{m=1}^{M}\sum_{i=1}^{N}v_{i}^{k}{\left(s_{i}-r_{i,m}\right)}^{2}\right)\;\;,\\ \text{such}\;\text{that} & & w_{ij}^{k}=\left\{\begin{array}{cc} 1 & \text{if}\;r_{i,k}\neq -\;\text{and}\;r_{j,k}\neq -\\ 0 & \text{otherwise} \end{array}\right.\;\;\text{and}\;\;v_{i}^{k}=\left\{\begin{array}{cc} 1 & \text{if}\;r_{i,k}\neq -\\ 0 & \text{otherwise} \end{array}\right.\;\;, \end{array}$$ (4)

where *α* is a positive real number that weights how much the first part of the objective function (the *separation* penalty) is relatively important with respect to the second part (the *deviation* penalty).

[31] and [32] discuss how their SD method, from Equation (4), can be solved in *O*(*MN*log(*N* ^2^/*M*)log*N*) time, constructing first an *NM* × *NM* adjacency matrix, and then applying the *minimum cut* problem to the network resulting from that matrix, with the algorithm proposed by [34].

Comparing the two computational times *O*(*M* ^2.373^) (our method) and *O*(*MN*log(*N* ^2^/*M*)log*N*) (the SD method), it is clear that when *N* is large (and it may easily exceed *M* ^1.373^ in the online applications that we have in mind), then our method is computationally more efficient than the SD one. Also, in terms of memory storage, we deal with an *aM* × *aM* matrix while the SD method needs to store an *NM* × *NM* adjacency matrix. Again, a large *N* makes the SD method unfeasible.

### 2.5 Objective measures

How do we compare different rating methods? Any method that aggregates the score from an *N* × *M* matrix of marks will result in an *M* vector $\vec{s}$ ∈ [1, *a*]^*M*^, where [1, *a*] is the set of real numbers in–between 1 and *a*. When many data in the *N* × *M* matrix **r** of marks are missing, simple correlation between this vector $\vec{s}$ and the rows of the matrix are ambiguously defined and difficult to interpret.

First of all, we adopt the objective measure of the SD method from Equation (4), where we impose *α* = 1, calling it *SD measure*. This optimization problem is not trivial, but its solution is obtained from a system of linear equations, so it is generally unique. However, this measure has a huge variance across different random realizations of matrix **r**. As we have checked in the simulations (see Section 3 below), the value of this objective function computed in the optimum and the value computed on the simple average are not different with statistical significance over random realizations of matrix **r**. Also, when we apply this measure to real data in Section 4 we observe that any method does not differ more than 1% from any other with respect to this measure.

The measure that we use to evaluate the performance of the result $\vec{s}$ is the Kendall’s Tau, as used in [35], where the relation between $\vec{s}$ and ${\vec{r}}_{k}$, on each couple of items *i* and *j*, is given by the following formula:

Cij=0.5if(rk,i=`-')or(rk,j=`-')otherwise1if(si<rk,iandsj<rk,j)or(si>rk,iandsj>rk,j)or(si=rk,iandsj=rk,j)0.5if(si=rk,iandsj≠rk,j)or(si≠rk,iandsj=rk,j)0otherwise.

The Kendall Tau correlation between $\vec{s}$ and ${\vec{r}}_{k}$ is given by

$$\begin{array}{c} \tau_{k}=4\frac{\sum_{i=1}^{M-1}\sum_{j=i+1}^{M}C_{ij}}{M(M-1)}-1, \end{array}$$ (5)

which lies always between −1 and 1. And finally, the aggregate Kendall Tau correlation between $\vec{s}$ and **r** is given by the average $\tau =\sum_{k=1}^{N}\tau_{k}/N$. The analogy of this measure with the SD objective function shown in Equation (4) are that absent marks have weight 0 in the overall computation process. However, it differs in two ways: firstly this measures increases with higher correlation; secondly, it is only an *ordinal* measure, in the sense that only the ordering between numbers is important. We show in next section, when presenting the outputs of our simulation exercise, that this measure has the necessary stability that guarantees identification of better methods.

We end this section with an example.

### 2.6 Example

**Fig 2:**
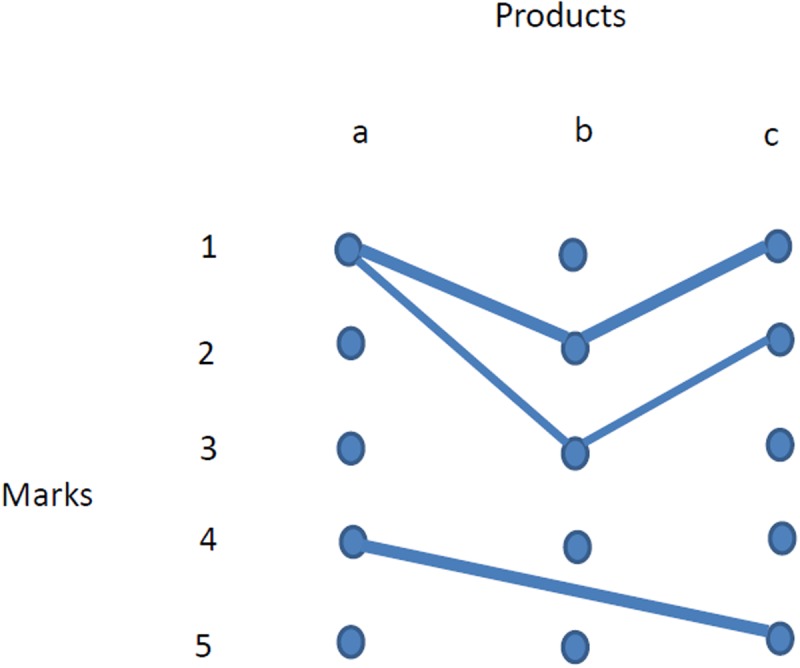
The network representation of Table 3 (a bolder line means higher weight).

Consider a simple case of three items and four users, depicted by Table 3. In this case, averaging results in values (2, 2.33, 2.67) assigned to the items, and this conflicts with the rankings of users 2 and 4. A ranking that instead values item 2 in the first place, then item 3 and finally item 1 would coincide with the order of each user. If we look at the network representation of Fig. 2, analogous to the one in Fig. 1 (where the weight of each link is given by formula (1)), we see that user 1 represents just an isolated component of the network, so that its scores have little in common with the other users.

**Table 3:** Table for Example 2.6.

	*Users*			
*Items*	1	2	3	4
*a*	4	–	1	1
*b*	–	2	2	3
*c*	5	1	–	2

Table 4 shows the outcome of the SD method and of the simple averaging, with respect to our NC method, with the values of *β* listed in Section 2.3 (in this example *λ* ^+^ is 1.560 and *N* = 4). The first three lines display the aggregated score for each of the three items: it is clear from here that as *β* → 0 we asymptotically approximate the average. The last two lines report the SD measure and the Kendall’s Tau of each method. The NC method with *β* = 2/(*λ* ^+^ + *N*) is the second best compared to the SD measure (which is the measure that the SD method minimizes by definition). The two NC methods with higher *β* values are also those that preserve the correct ordering implied by the users, as shown by the Kendall’s Tau. This example anticipates the results that we obtain from simulations and from an application to real data in Sections 3 and 4: the NC method with *β* = 2/(*λ* ^+^ + *N*) performs almost as well as the SD method, being computationally more efficient.

**Table 4:** Different methods applied to the example of Table 3.

			*β*						
*products*	SD	Avg	1/*λ* ^+^	$\frac{2}{\lambda^{+}+N}$	$\frac{1}{N}$	$\frac{1}{5N}$	$\frac{1}{10N}$	$\frac{1}{25N}$	$\frac{1}{50N}$
a	1.667	2.000	1.000	1.783	1.879	1.982	1.991	1.997	1.998
b	2.889	2.333	2.302	2.325	2.330	2.333	2.333	2.333	2.333
c	2.444	2.667	1.403	2.322	2.460	2.632	2.650	2.660	2.663
*SD*_*measure*_	18.111	22.380	24.438	20.580	21.111	22.100	22.218	22.292	22.310
*τ*_*Kendall*_	0.5	0.1667	0.5	0.5	0.1667	0.1667	0.1667	0.1667	0.1667

## 3 Simulations

We test the quality of our method, with the values of *β* listed in Section 2.3, by comparing them with the SD method and with the simple averaging, on synthetic data generated in the following way. We consider *a* = 5 (so, five possible scores) and we vary the number of users from *N* = 10 (ten users) to *N* = 50 (fifty users). For *M*, the number of items, we have two cases: *M* = 10 and *M* = 50. Each user *i* rates randomly, with *i*.*i*.*d*. probabilities, in the following way: each item *j* is not rated with probability 1 − *p* (so that *r*_*i*, *j*_ = `−’) and rated with probability *p*. When rated, *r*_*i*, *j*_ has one of the 5 possible values from {1, 2, 3, 4, 5} with uniform probabilities. In this way, every single element of the matrix *r*_*i*, *j*_ is i.i.d. with respect of all the others, and a fraction 1 − *p* of them are expected to be empty: `−’. First, we analyze how the different rating methods perform with respect to the Kendall’s Tau correlation from Equation (5), in the four cases (*N*, *M*) = (10, 10), (*N*, *M*) = (10, 50), (*N*, *M*) = (50, 10) and (*N*, *M*) = (50, 50), showing that the results are not sensitive to size. Then, in a case with *N* = 30 and *M* = 50, we check for robustness of different measures when additional users with systematically biased reports are added into the sample, varying the way in which this bias is generated.

### 3.1 Kendall’s tau correlation

For the four cases (*N*, *M*) = (10, 10), (*N*, *M*) = (10, 50), (*N*, *M*) = (50, 10) and (*N*, *M*) = (50, 50) we generate 200 i.i.d. realizations of **r**, for 13 evenly spaced values of *p* in–between 0.4 (less than half of the items are expected to be rated by each user) and 1 (all items are rated by each user). For each realization we compute the average mark for each item, the measure resulting from the SD method of [32], and our centrality measure with respect to the 7 different values of *β* discussed in Section 2.3: largest eigenvalue *λ* ^+^ of the *L* matrix, 1/*N*, 2/(*λ* ^+^ + *N*), 1/5*N*, 1/10*N*, 1/25*N* and 1/50*N*. Finally, for each of these *M*–dimensional vectors of measures, we compute the Kendall’s Tau correlation from Equation (5).

**Fig 3:**
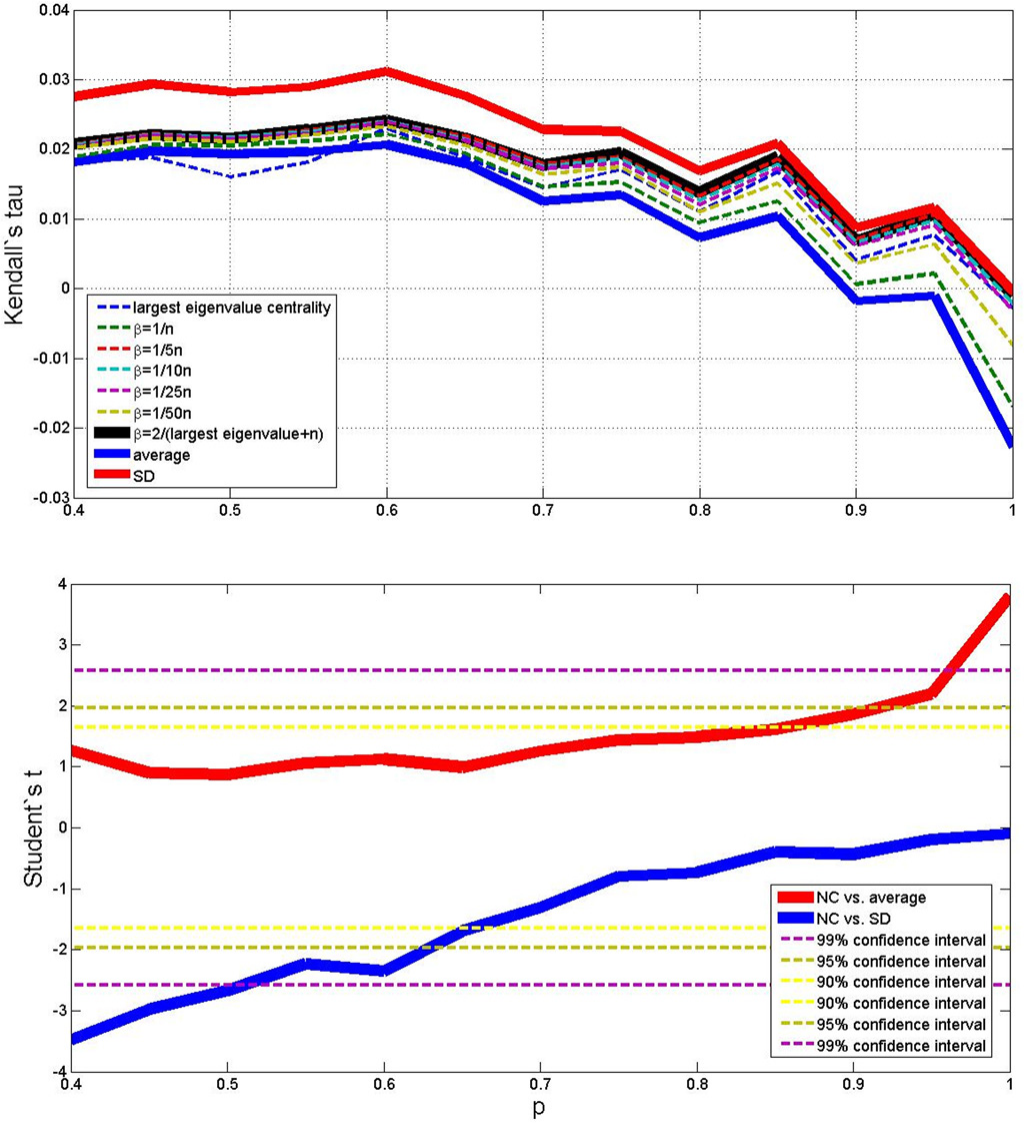
Average outcome of the Kendall’s tau measure on the simulations, with 9 different methods, *p* varying from 0.4 to 1, *N* = 10 and *M* = 10. Lower plot shows Student’s *t* comparison of the NC method with *β* = 2/(*λ* ^+^ + *N*) with respect to average and SD—confidence intervals are shown in the legend.

**Fig 4:**
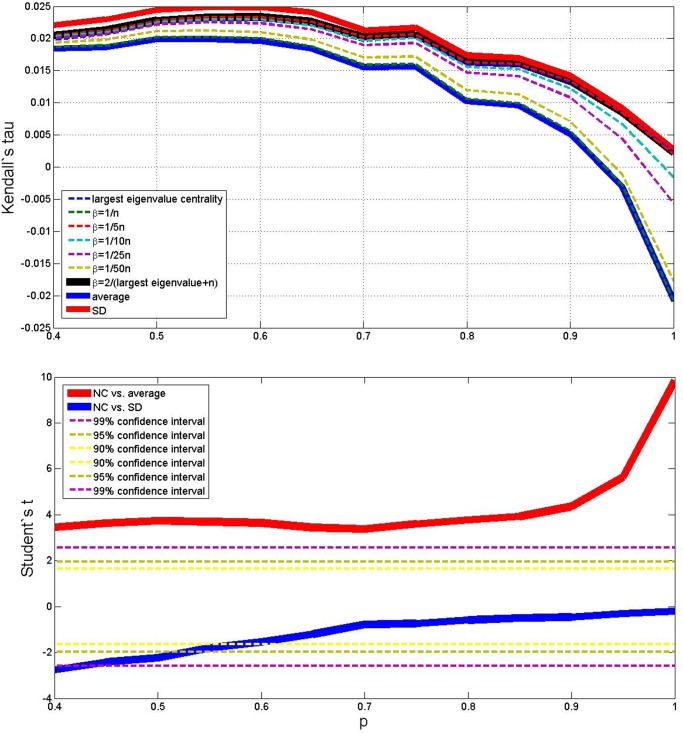
Average outcome of the Kendall’s tau measure on the simulations, with 9 different methods, *p* varying from 0.4 to 1, *N* = 10 and *M* = 50. Lower plot shows Student’s *t* comparison of the NC method with *β* = 2/(*λ* ^+^ + *N*) with respect to average and SD—confidence intervals are shown in the legend.

**Fig 5:**
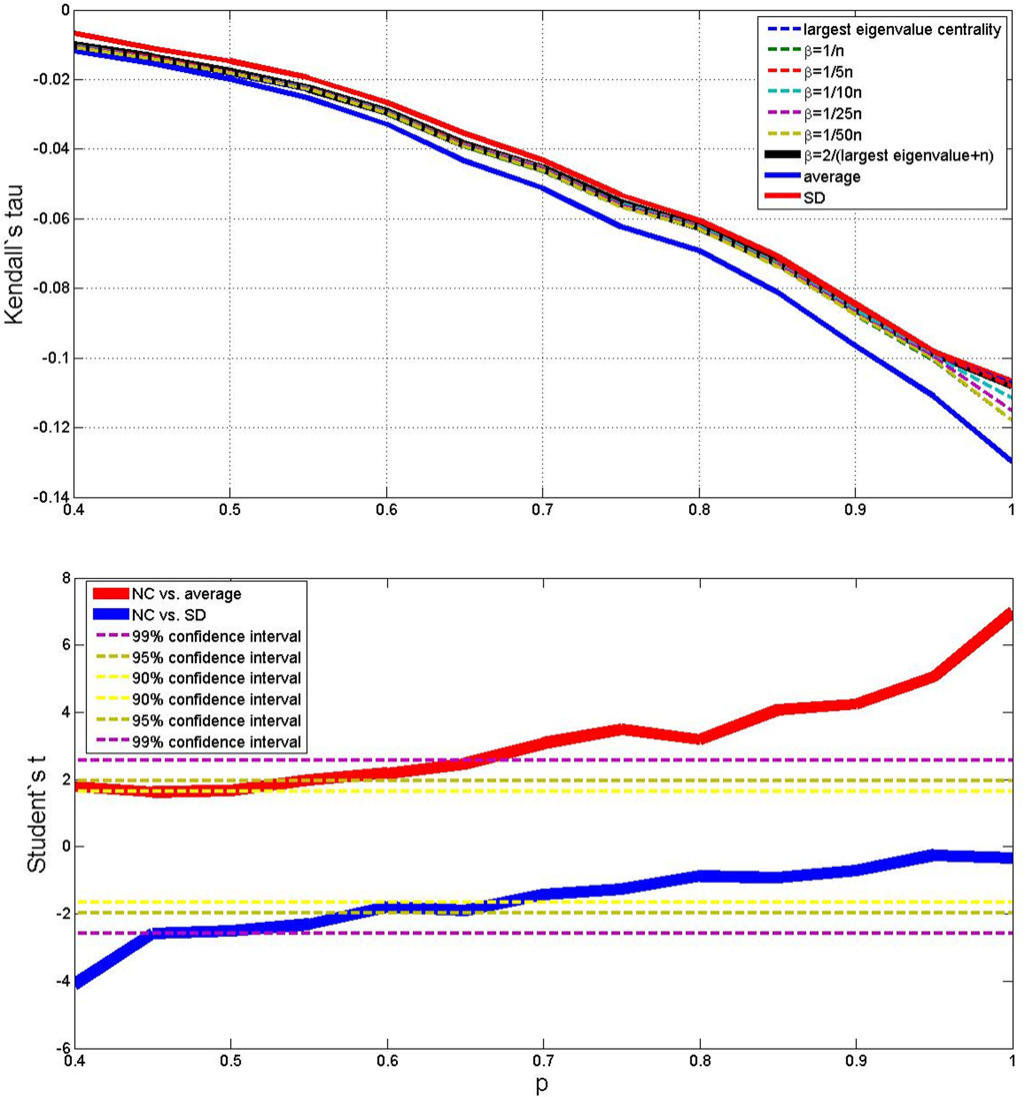
Average outcome of the Kendall’s tau measure on the simulations, with 9 different methods, *p* varying from 0.4 to 1, *N* = 50 and *M* = 10. Lower plot shows Student’s *t* comparison of the NC method with *β* = 2/(*λ* ^+^ + *N*) with respect to average and SD—confidence intervals are shown in the legend.

**Fig 6:**
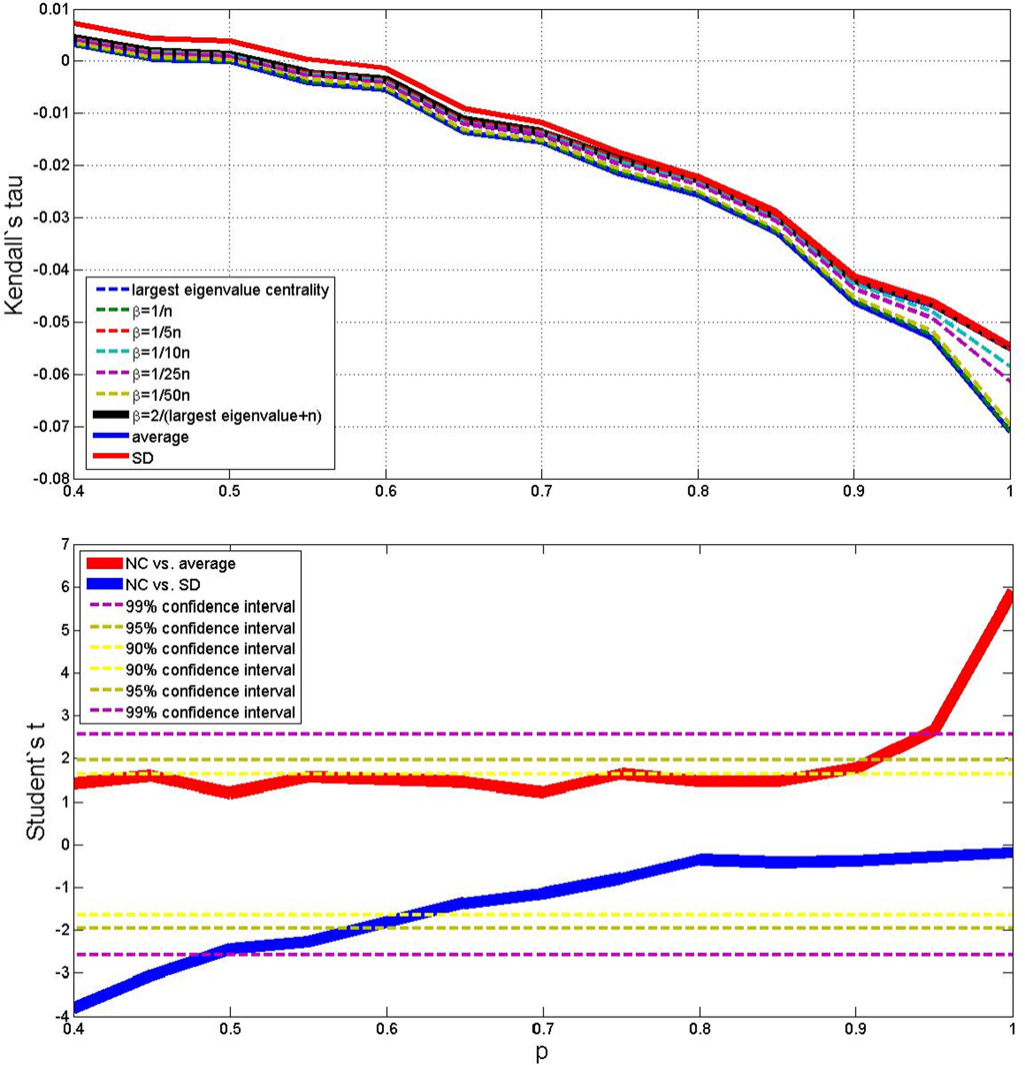
Average outcome of the Kendall’s tau measure on the simulations, with 9 different methods, *p* varying from 0.4 to 1, *N* = 50 and *M* = 50. Lower plot shows Student’s *t* comparison of the NC method with *β* = 2/(*λ* ^+^ + *N*) with respect to average and SD—confidence intervals are shown in the legend.

Results of the average outcomes for the four cases are reported in the upper parts of Figs. 3, 4, 5 and 6. From these average trends, it comes out clearly out that SD is the best performing measure (but it is more costly than the NC method both in terms of computational time and memory storage), and that averaging is the worse, while the centrality measure with different values of *β* lies in–between. The best value of *β* seems to be 2/(*λ* ^+^ + *N*). However, we need to take variance into account when analyzing these results. In the Supporting Information, Figures S1 to S4 display the boxplots of all the 200 realizations, for each of the four cases, of the following three measures: DS, average, and NC measure with *β* = 2/(*λ* ^+^ + *N*). The lower parts of Figs. 3 to 6 take also variance into account and plot the Student’s *t*–test to check whether the centrality measure with *β* = 2/(*λ* ^+^ + *N*) is statistically different from the SD measure and the simple average, as *p* changes. When (*N*, *M*) = (10, 10), (*N*, *M*) = (50, 10) and (*N*, *M*) = (50, 50) (Figs. 3, 5 and 6) the NC measure is not statistically different from the other two measures for most values of *p*: it performs significantly better than the average for high *p*, and significantly worse than the MS measure for low *p*. But when (*N*, *M*) = (10, 50) (Fig. 4), the NC method is always better than the average with 99% statistical confidence, while it is not statistically different form the MS measure for *p* above 0.6.

We have tried, on the same set of simulations, also two other measures of coherence: the objective measure from Equation (4) and the simple average correlation of a method with those of all the users (limited for each user to those goods that are actually rated by that user). However, those two measures have a much larger variance than the Kendall’s tau correlation, and all the outputs are not statistically different, according to the Student’s *t*–test, in any of the points presented in Figures S1 to S4. The reason is clearly that while Kendall’s tau correlation is penalized only by violations in the ordering, and is hence *ordinal*, the measure from Equation (4) and the simple correlation are cardinal measures that are affected also by the magnitude of the scores.

### 3.2 Robustness to biased fake data

Now we pose a different question: what happens to the ranking measure that we are using if we add fictitious users who adopt a systematically biased report? To do so we consider the previous case with *N* = 30 and *M* = 50, and we add *H* users (with *H* from 3 to 15) to the original 30 ones. These users just assign mark 1 to the first three items in the *M* list, and mark 5 to the last three ones. It is clear that the order of the items plays no role, and the point is just that some goods are systematically rated at the top, while others are systematically rated at the bottom. What is important here is that the two bias balance each others on average, so that we would expect the simple average ranking to perform well in this scenario. In principle, a good measure should detect that the fictitious fake users are somehow *different* from the original ones. The NC measure does exactly this, because the sub–network generated by the new fictitious users will be an almost disconnected part with respect to the original network ([12] and [36] propose algorithms to detect fake accounts, also knows as *Sybils*, that are based on the same intuition that sybils’ behavior is unrelated with the ranking activity of real users—clearly this works only as long as the number of those fake users is *small* with respect to the number of real users).

**Fig 7:**
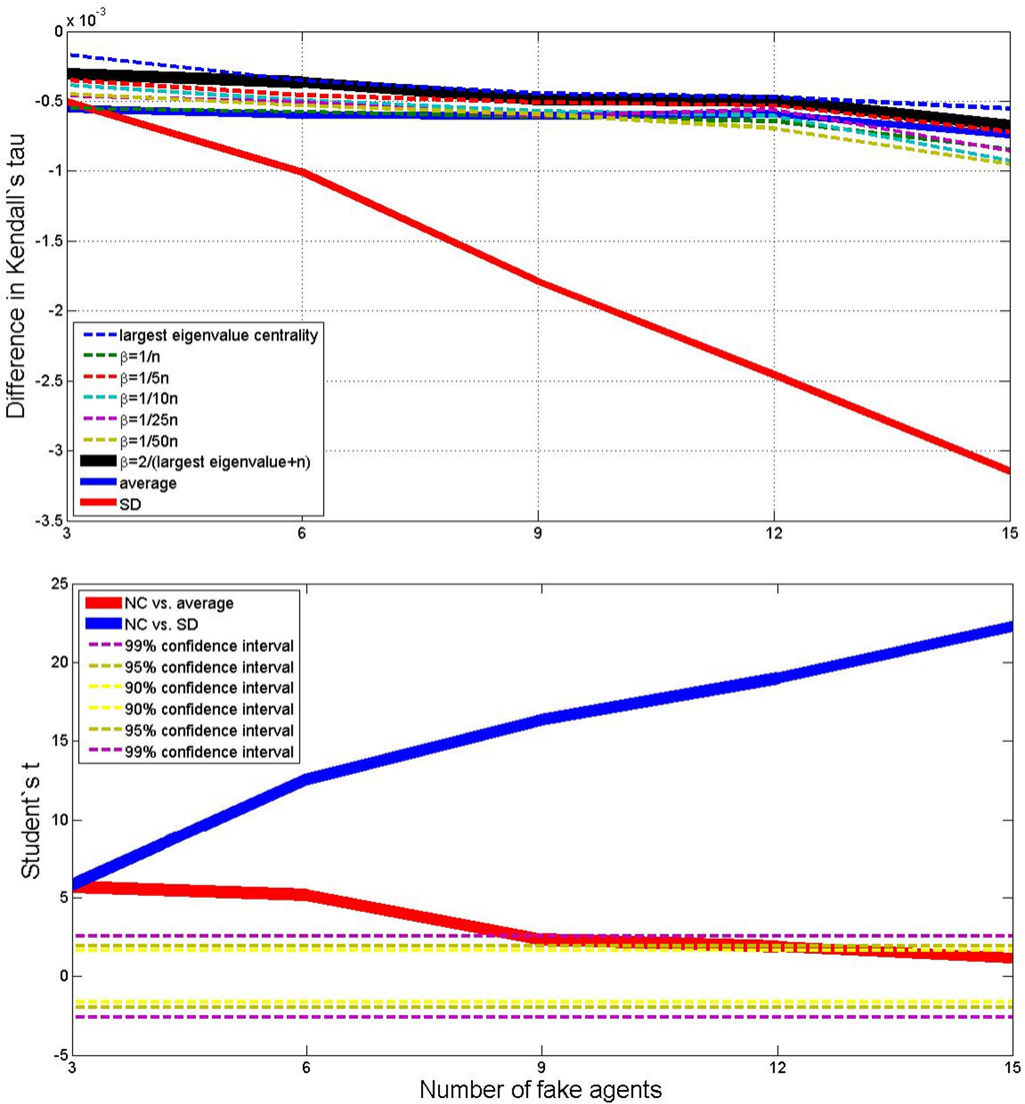
Average outcome in the simulations of the difference in Kendall’s tau measure, between the original data and those obtained by adding *X* fake. There are 9 different methods, and *X* varies from 1 to 5. Lower plot shows Student’s *t* comparison of the NC method with *β* = 2/(*λ* ^+^ + *N*) with respect to average and SD—confidence intervals are shown in the legend.

As a measure for our simulations we use the difference between the Kendall’s tau correlation index computed on the measure obtained by aggregating all the users, and the Kendall’s tau correlation index computed on the measure that would have been obtained only from the original users. Clearly, this difference will be negative, but a good measure will be one that minimizes it in absolute value. With the same notation the figures in the last section, Fig. 7 reports the outcome and the *t*–test (and Figure S5 in the Supporting Informations shows the boxplots).

The results speak in great favor of our NC method: when up to 12 fake users are added to the original 30 ones, the NC measure with *β* = 2/(*λ* ^+^ + *N*) is better than any other measure with a high statistical significance. When even more fake users are added it further improves its outcome compared to the SD measure, but it becomes not signifcantly better than the average.

## 4 Real data

Finally, we test our method, with the values of *β* listed in Section 2.3, with respect to the SD method and to the simple average, on a real dataset. In this case we consider all measures of efficiency, but since it is a single realization we will not be able to compute statistical significance when the outcomes are different.

We use a dataset recording rating information on movies provided by the site grouplens.org, from the University of Minnesota [33]. The data set encompasses 943 users and 1682 movies. It records the rating scores of each movie rated by its users. All the movies are rated from score 1 to score 5, with 1 as the worst and 5 as the best. The total amount of rating records are about 100,000, so each customer rated on average about 106 movies which is far less than 1,682. Such sparse data would pose a challenge for many of the existing evaluation methods. Also, the SD method would need to construct a matrix of size (*NM*)^2^ > 2.5 ⋅ 10^12^, in order to apply the minimum cut algorithm of [34] (we have computed directly with *Matlab* the direct optimization of the objective function shown in formula (4)). For our NC method we need instead to invert a matrix of size (5*M*)^2^ ≃ 7 ⋅ 10^7^, which is tractable.

The results, reported in Table 5, are consistent with those of the simulations. The NC method with *β* = 2/(*λ* ^+^ + *N*) performs almost as well as the SD method with respect to the Kendall’s tau correlation, and we know that it is computationally more efficient. It is surprising that the SD method is actually the worst one with respect to the simple Pearson correlation, but we have discussed in Section 2.5 how it is difficult to interpret correlation when many missing data are present, as in this case. Finally, the SD method, by definition minimizes the objective function from Equation (4), and with this respect the NC method is not better than the simple average, even if all the numbers are very similar and there is no clear added value in not adopting the simple average. Actually, in relative terms the difference between the NC method with *β* = 1/*λ* ^+^ and the SD method is about 0.5%.

**Table 5:** Different methods adopted on the real dataset.

‘Movielens’ dataset			
Method	*τ*_*Kendall*_ ⋅ 10^−5^	Correlation	SD value ⋅ 10^7^
*β* = 1/*λ* ^+^	0.48	0.4256	1.699964
*β* = 2/(*λ* ^+^ + *N*)	0.61	0.4208	1.692521
*β* = 1/5*N*	0.55	0.4205	1.692400
*β* = 1/10*N*	0.53	0.4202	1.692308
*β* = 1/25*N*	0.52	0.4202	1.692302
*β* = 1/50*N*	0.52	0.4202	1.692294
Average	0.44	0.4202	1.692244
SD	0.63	0.4099	1.691011

## 5 Conclusion

We have provided a network centrality rating method for aggregating the overall information of users rating items. We argue that it is an optimal trade–off between computational efficiency and desirable features, especially when compared with the simple average and with other methods proposed in the literature. However, the methods actually used by online sites are not evident, and consumers can only perceive them as a black–box. The algorithms implemented by those online sites are probably very sophisticated, and make use of many more information than those we have considered in the present paper. For example, the popular site www.tripadvisor.com, which is actually based only on its rating service, declares that it gives different weights to different reports depending on the *importance* of the reviewer and on the timing, attributing higher value to more recent reports (this is for example explicitly stated in the following two urls: http://help.tripadvisor.com/articles/200613987 and http://help.tripadvisor.com/articles/200614027). The service of aggregating ratings could be also customized upon request, as the *importance* could be assigned in a way that reviewers with certain characteristics are given more or less weights depending on who performs the request (this activity falls in the domain of *recommender systems*). What we want to stress here is that attributing different weights to consumers or reports is something that can easily be done in any of the methods we have compared with, including the average and our NC method. Actually, showing that at the limit of *β* → 0 our NC method actually coincides with the average, we provide continuity between any weighted average method and the corresponding NC method with *β* > 0. Thus, the *horse–race* exercise that we have performed would not be affected by this extra differentiation.

Also, we have shown that our NC method is, in some cases, the best one in neglecting the score of systematically biased fake reports. This works as long as those fake users constitute a minority of the population and behave systematically in a way that is disconnected from the rating activity of real users. It is clear that knowing who this fake and/or biased users are will help in disregarding their value, as one could give them less or even zero weight, but our NC method does it generically by its own nature, starting ex–ante with equal weights and no extra information.

Besides, any other additional information or algorithm that can distinguish the importance of users and even single reports will be helpful for our method, as for any other one, but it is orthogonal to the properties that we have shown in the present paper. In this sense, we provide an additional tool that can be implemented in real world applications, and we show that its properties are very useful in obtaining an aggregate rating measure that preserves the original rankings implide by users, and is able to detect, without any additional information, those users who are under suspicion of being not genuine.

## Supporting Information

S1 File **Fig. A in S1 File** Full boxplot of the simulation summarized in Fig. 3, for the average, the SD method, and the NC method with *β* = 2/(*λ* ^+^ + *N*).
**Fig. B in S1 File** Full boxplot of the simulation summarized in Fig. 4, for the average, the SD method, and the NC method with *β* = 2/(*λ* ^+^ + *N*).
**Fig. C in S1 File** Full boxplot of the simulation summarized in Fig. 5, for the average, the SD method, and the NC method with *β* = 2/(*λ* ^+^ + *N*).
**Fig. D in S1 File** Full boxplot of the simulation summarized in Fig. 6, for the average, the SD method, and the NC method with *β* = 2/(*λ* ^+^ + *N*).
**Fig. E in S1 File** Full boxplot of the simulation summarized in Fig. 7, for the average, the SD method, and the NC method with *β* = 2/(*λ* ^+^ + *N*).
(PDF)

## References

[1] Ba S, Pavlou PA. Evidence of the effect of trust building technology in electronic markets: Price premiums and buyer behavior. MIS Quarterly 2002;26(3): 243–268. 10.2307/4132332

[2] Pavlou PA, Gefen D. Building effective online marketplaces with institution-based trust. Information Systems Research. 2004;15(1): 37–59. 10.1287/isre.1040.0015

[3] Pavlou PA, Liang H, Xue Y. Understanding and mitigating uncertainty in online exchange relationships: A principal-agent perspective. MIS Quarterly. 2007;31(1): 105–136.

[4] Park D–H, Kim S. The effects of consumer knowledge on message processing of electronic word-of-mouth via online consumer reviews.Electronic Commerce Research and Applications. 2009;7(4): 399–410. 10.1016/j.elerap.2007.12.001

[5] Resnick P, Iacovou N, Suchak M, Bergstrom P, Riedl J. Grouplens: an open architecture for collaborative filtering of netnews. In Proceedings of the 1994 ACM Conference on Computer Supported Cooperative Work, ACM. 1994: 175–186. 10.1145/192844.192905

[6] Sarwar B, Karypis G, Konstan J, Riedl J. Item-based collaborative filtering recommendation algorithms. In Proceedings of the 10th International Conference on World Wide Web, ACM. 2001: 285–295.

[7] Adomavicius G, Tuzhilin A. Toward the next generation of recommender systems: A survey of the state-of-the-art and possible extensions. Knowledge and Data Engineering, IEEE Transactions. 2005;17(6):734 –749. 10.1109/TKDE.2005.99

[8] Markines B, Cattuto C, Menczer F, Benz D, Hotho A, Stumme G. Evaluating similarity measures for emergent semantics of social tagging. In Proceedings of the 18th International Conference on World Wide Web, ACM. 2009: 641–650. 10.1145/1526709.1526796

[9] Trevisiol M, Chiarandini L, Aiello LM, Jaimes A. Image ranking based on user browsing behavior. In Proceedings of the 35th International ACM SIGIR Conference on Research and Development in Information Retrieval ACM. 2012: 445–454.

[10] Arrow KJ. Social Choice and Individual Values. Yale University Press; 1963.

[11] Basili M, Pratelli L. Aggregation of not independent experts’ opinions under ambiguity. Structural Safety. 2014;52(B): 144–149.

[12] Wang G, Mohanlal M, Wilson C, Wang X, Metzger M, Zheng H, Zhao BY. Social turing tests: Crowdsourcing sybil detection. In Proceedings of the Network and Distributed System Security Symposium (NDSS 2013). 2013.

[13] Cao Q, Sirivianos M, Yang X, Pregueiro T. Aiding the detection of fake accounts in large scale social online services. In Proceedings of the 9th USENIX Conference on Networked Systems Design and Implementation, USENIX Association. 2012: 15–15.

[14] Wang G, Wilson C, Zhao X, Zhu Y, Mohanlal M, Zheng H, Zhao BY.Serf and turf: crowdturfing for fun and profit. In Proceedings of the 21st International Conference on World Wide Web, ACM. 2012: 679–688. 10.1145/2187836.2187928

[15] Girotra K, Terwiesch K, and Ulrich KT. Idea generation and the quality of the best idea. Management Science. 2010;56(4): 591–605. 10.1287/mnsc.1090.1144

[16] Bonacich P, Lloyd P. Eigenvector-like measures of centrality for asymmetric relations. Social Networks. 2001;23(3): 191–201. 10.1016/S0378-8733(01)00038-7

[17] Newman MEJ. The Structure and Function of Complex Networks. SIAM Review. 2003;45: 167–256. 10.1137/S003614450342480

[18] Jackson MO. Social and Economic Networks. Princeton University Press; 2008.

[19] Boldi P, Vigna S. Axioms for centrality. Internet Mathematics. 2014;(3–4): 222–262. 10.1080/15427951.2013.865686

[20] Newman MEJ. Analysis of weighted networks. Physical Review E. 2004;70 (5): 056131. 10.1103/PhysRevE.70.056131 15600716

[21] Opsahl T, Agneessens F, Skvoretz J. Node centrality in weighted networks: Generalizing degree and shortest paths. Social Networks. 2010;32(3): 245–251. 10.1016/j.socnet.2010.03.006

[22] It is also possible to assign centrality studying the spectral analysis of *L* and assign weights with the eigenvectors corresponding to larger eigenvalues. However, as pointed out in [16], this is an approach that is much less robust to perturbations.

[23] Banerjee A, Chandrasekhar AG, Duflo E, Jackson MO. Gossip: Identifying central individuals in a social network. Technical report, National Bureau of Economic Research. 2014.

[24] Bramoullé Y, Kranton R, D’Amours M. Strategic interaction and networks. The American Economic Review. 2014;104(3): 898–930. 10.1257/aer.104.3.898

[25] Actually, the Perron–Frobenius theorem states only that λ^+^ is the unique strictly positive eigenvalue. Consider now that the sum of the elements in each row of column *xi* is the number of users who actually rated good *x* with score *i*, and this number is clearly bounded above by the total number *N* of users. So, any eigenvalue of the matrix (that is how much the corresponding eigenvector is multiplied in the matrix product) cannot exceed *N* in absolute value.

[26] Williams VV. Multiplying matrices faster than Coppersmith-Winograd. In Proceedings of the 44th Symposium on Theory of Computing, ACM 2012: 887–898.

[27] Amestoy PR, Duff IS, L’Excellent J–Y, Robert Y, Rouet F–H, Uçar B. On computing inverse entries of a sparse matrix in an out-of-core environment. SIAM Journal on Scientific Computing. 2012;34(4): A1975–A1999. 10.1137/100799411

[28] Saaty TL. A scaling method for priorities in hierarchical structures. Journal of Mathematical Psychology. 1977;15(3): 234–281. 10.1016/0022-2496(77)90033-5

[29] Keener JP. The Perron-Frobenius theorem and the ranking of football teams. SIAM Review. 1993;35(1): 80–93. 10.1137/1035004

[30] Kemeny JG, Snell LJ. Preference ranking: an axiomatic approach. Mathematical Models in the Social Sciences. 1962: 9–23.

[31] Hochbaum DS, Levin A. Methodologies and algorithms for group-rankings decision. Management Science. 2006;52(9): 1394–1408. 10.1287/mnsc.1060.0540

[32] Hochbaum DS, Moreno–Centeno E, Yelland P, Catena RA. Rating customers according to their promptness to adopt new products.Operations Research. 2011;59(5): 1171–1183. 10.1287/opre.1110.0963

[33] The actual dataset is called *movielens* and is part of an academic project of the University of Minnesota on *Social Computing*. ‘Movielens’ is presented as a tool for rating and comparing movies: anyone can register, rate and consult all the movies from a fixed list. The version we analyze has been downloaded by us in December 2013.

[34] Ahuja RK, Hochbaum DS, Orlin JB. Solving the convex cost integer dual network flow problem. Management Science. 2003;49(7): 950–964. 10.1287/mnsc.49.7.950.16384

[35] Vanhoucke M. Using activity sensitivity and network topology information to monitor project time performance. Omega. 2010;38(5): 359–370. 10.1016/j.omega.2009.10.001

[36] Yang Z, Wilson C, Wang X, Gao T, Zhao BY, Dai Y. Uncovering social network sybils in the wild. ACM Transactions on Knowledge Discovery from Data (TKDD). 2014;8(1): 2 10.1145/2556609

