# Supplementary material for: A Network Centrality Method for the Rating Problem
Source: PLoS One. 2015 Apr 1;10(4):e0120247. doi: 10.1371/journal.pone.0120247 (PMC4382193; doi:10.1371/journal.pone.0120247)
Supplement: S1 File — Fig. A in S1 File Full boxplot of the simulation summarized in Fig. 3, for the average, the SD method, and the NC method with β = 2/(λ + + N). Fig. B in S1 File Full boxplot of the simulation summarized in Fig. 4, for the average, the SD method, and the NC method with β = 2/(λ + + N). Fig. C in S1 File Full boxplot of the simulation summarized in Fig. 5, for the average, the SD method, and the NC method with β = 2/(λ + + N). Fig. D in S1 File Full boxplot of the simulation summarized in Fig. 6, for the average, the SD method, and the NC method with β = 2/(λ + + N). Fig. E in S1 File Full boxplot of the simulation summarized in Fig. 7, for the average, the SD method, and the NC method with β = 2/(λ + + N). (PDF) [file pone.0120247.s001.pdf]

Supporting informtion for
“A network centrality method for the rating problem”

Yongli Li[*] Paolo Pin [†] Chong Wu[‡]

February 2015

Boxplots from the simulations

For the five sets of simulations discussed in Section 3 of the Main Text, we report the boxplots of all the 200 outcomes on each set of variables, for the NC method with $\beta = 2/(\lambda^+ + N)$, the SD method, and the simple average. On the y–axis we report all the cases, and on the x–axis we report the measures: Kendall’s tau for the simulations discussed in Section 3.1 (Figures A to D) and the difference between Kendall’s tau measures, that we call *robustness*, for the simulation discussed in Section 3.2 (Figure E). It is clear that there is a large variance and many realizations overlap, and this is why we show in the figures of the main text also the outcome of Student’s t–tests to check when these outcomes are statistically different.

[*]School of Business Administration, Northeastern University, Shenyang 110819, P.R.China
[†]Dipartimento di Economia Politica e Statistica, Università degli Studi di Siena, 53100 Siena, Italy
[‡]School of Management, Harbin Institute of Technology, Harbin 150001, P.R.China

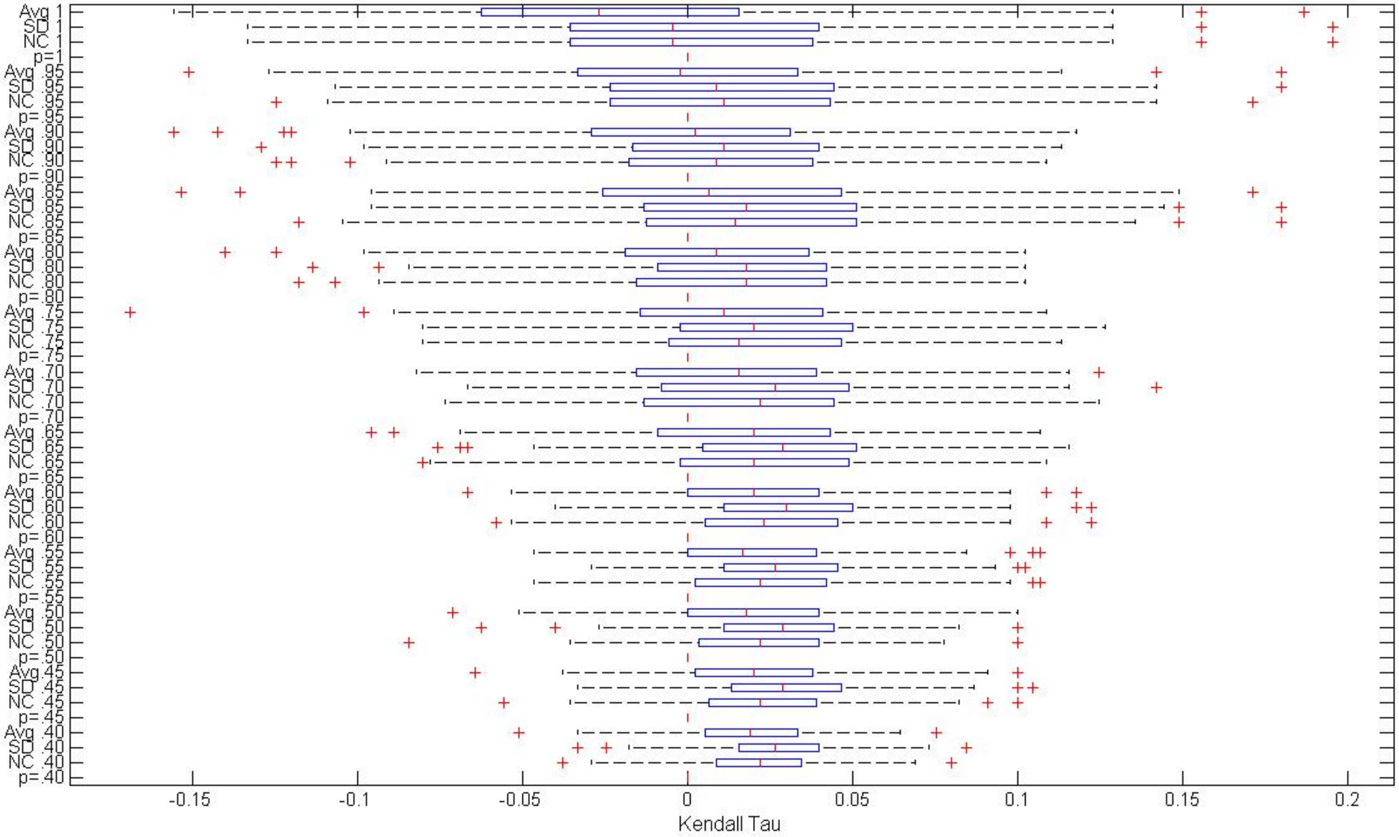

Figure A: Full boxplot of the simulation summarized in Figure 3 in the Main Text, for the average, the SD method, and the NC method with $\beta = 2/(\lambda^+ + N)$.

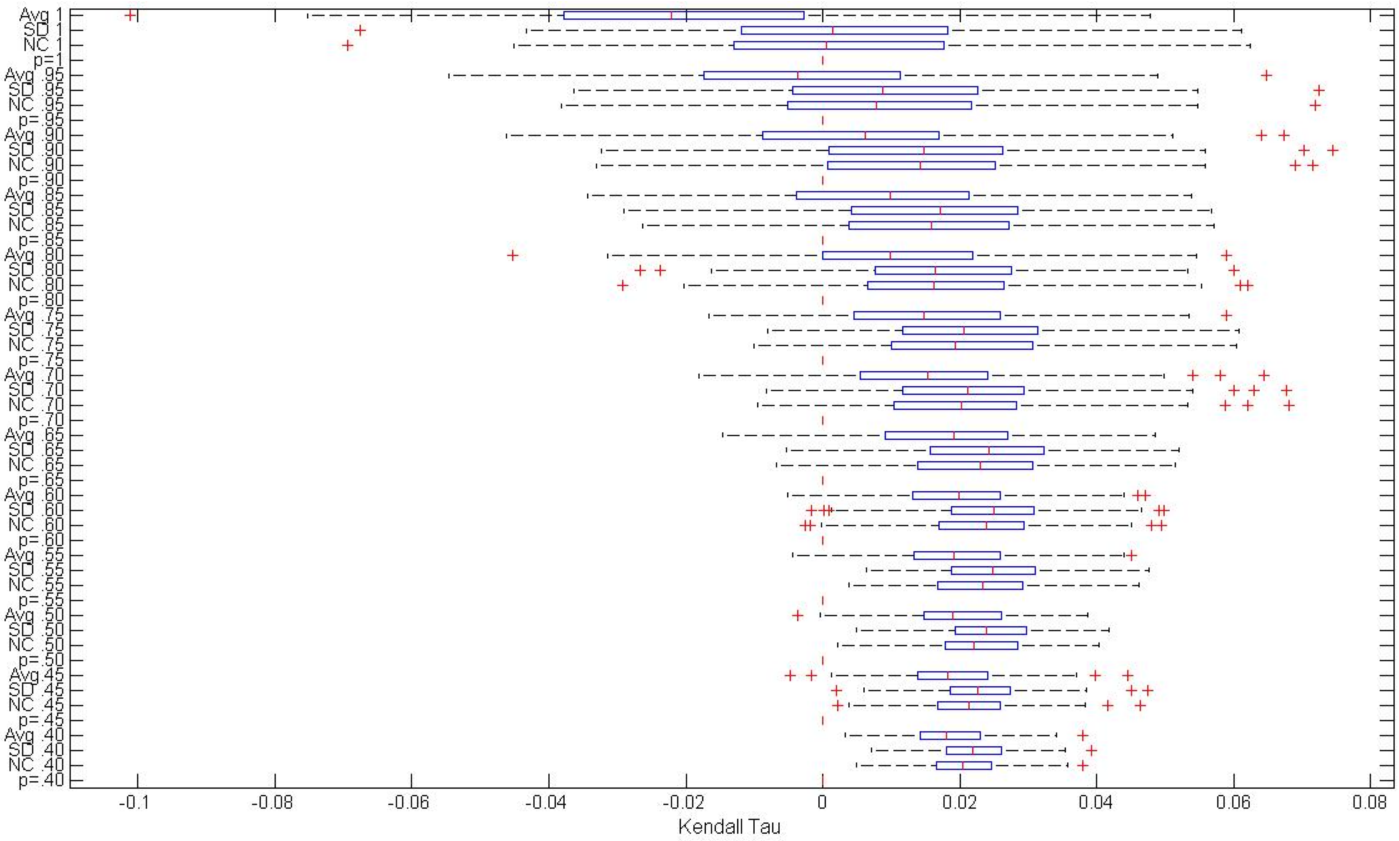

Figure B: Full boxplot of the simulation summarized in Figure 4 in the Main Text, for the average, the SD method, and the NC method with $\beta = 2/(\lambda^+ + N)$.

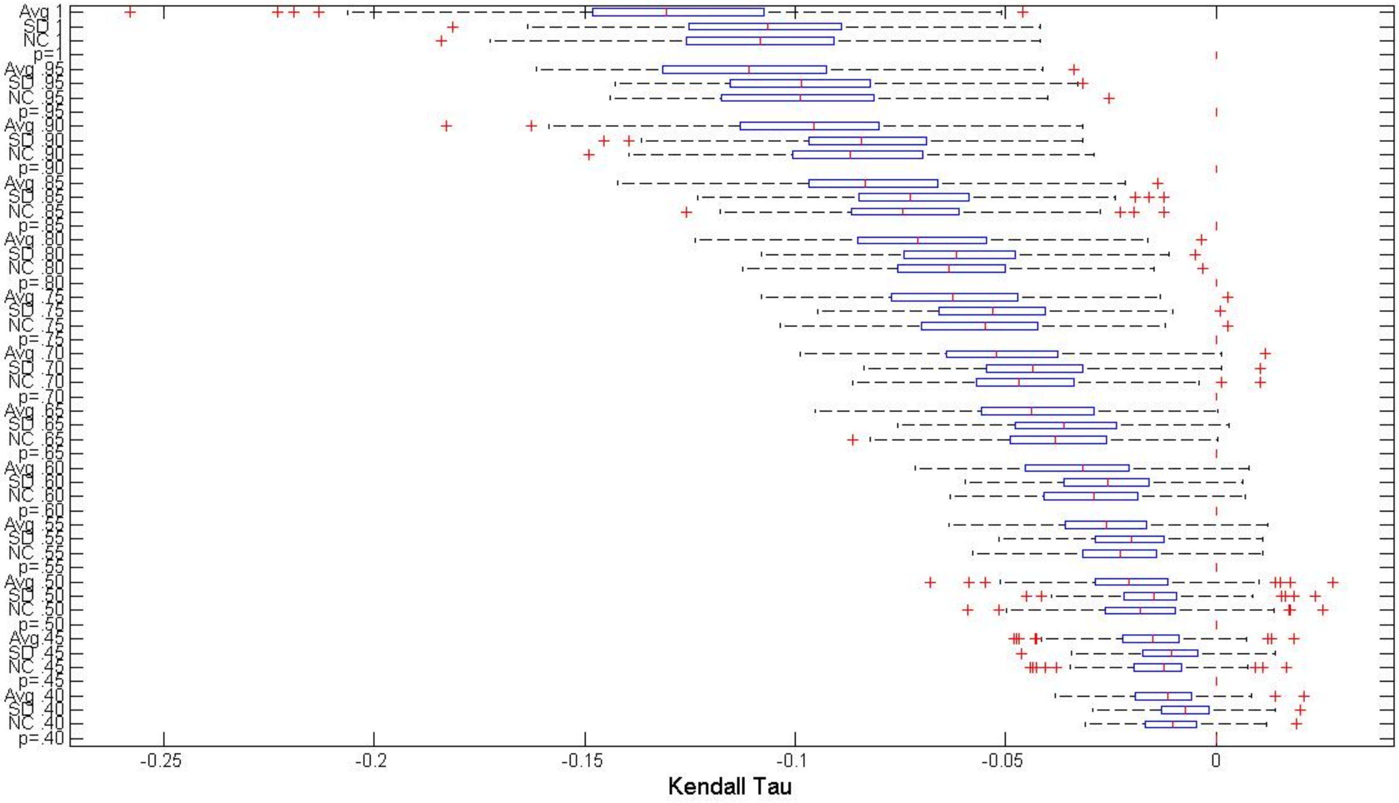

Figure C: Full boxplot of the simulation summarized in Figure 5 in the Main Text, for the average, the SD method, and the NC method with $\beta = 2/(\lambda^{+} + N)$.

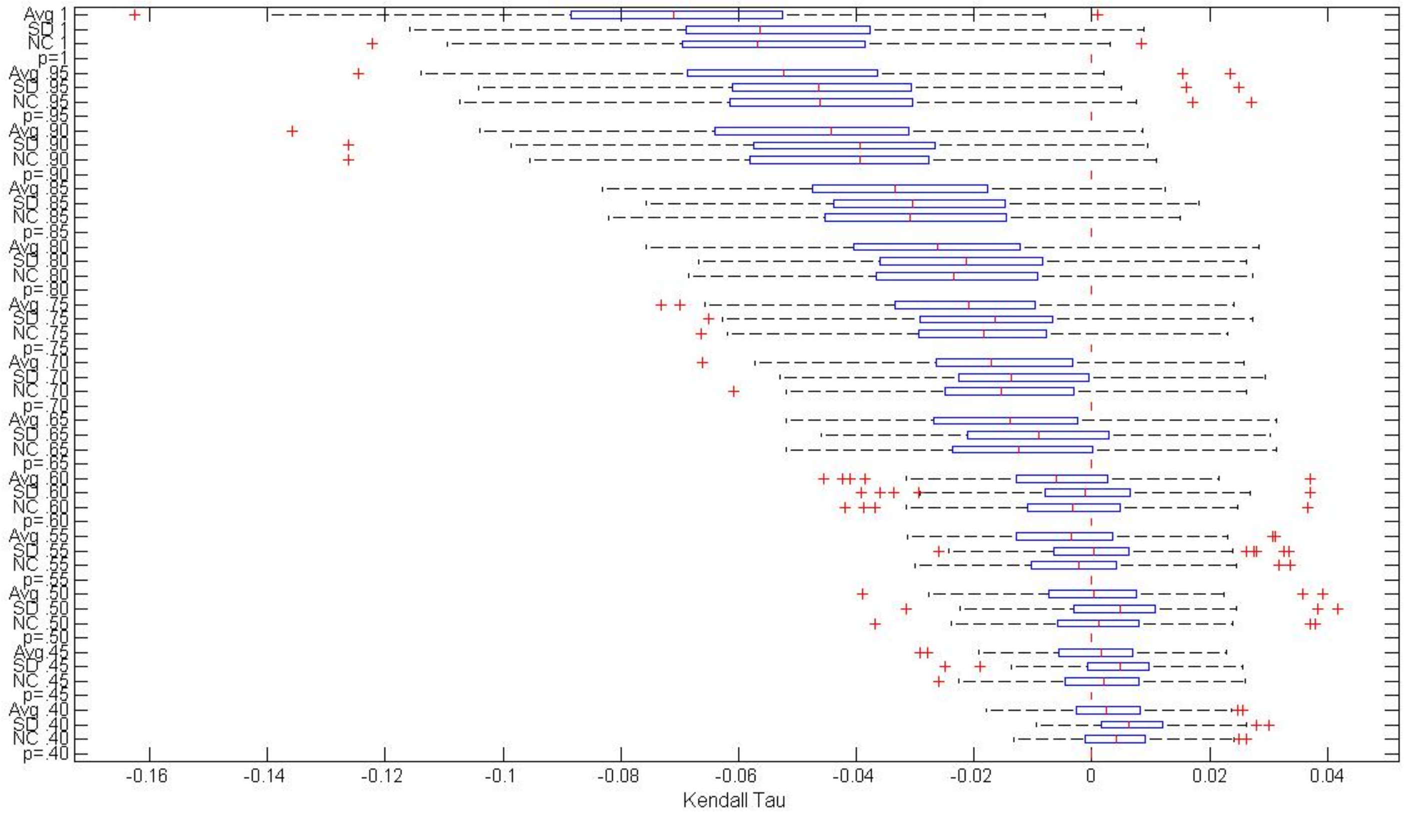

Figure D: Full boxplot of the simulation summarized in Figure 6 in the Main Text, for the average, the SD method, and the NC method with $\beta = 2/(\lambda^{+} + N)$.

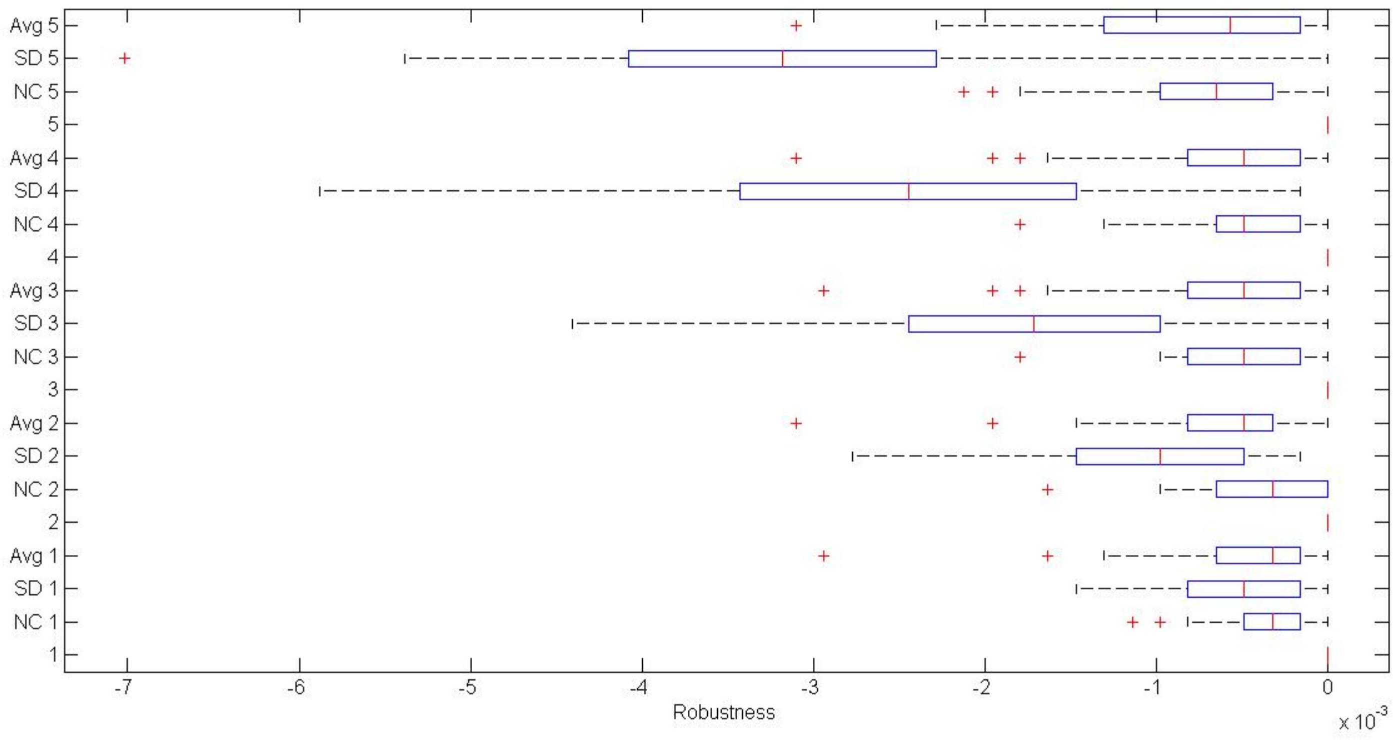

Figure E: Full boxplot of the simulation summarized in Figure 7 in the Main Text, for the average, the SD method, and the NC method with $\beta = 2/(\lambda^+ + N)$.
